# Genetic variants in taste genes play a role in oral microbial composition and severe early childhood caries

**DOI:** 10.1016/j.isci.2022.105489

**Published:** 2022-11-09

**Authors:** Vivianne Cruz de Jesus, Betty-Anne Mittermuller, Pingzhao Hu, Robert J. Schroth, Prashen Chelikani

**Affiliations:** 1 Manitoba Chemosensory Biology Research Group, Department of Oral Biology, University of Manitoba, Winnipeg, MB, Canada; 2 Children’s Hospital Research Institute of Manitoba (CHRIM), Winnipeg, MB, Canada; 3 Department of Preventive Dental Science, University of Manitoba, Winnipeg, MB, Canada; 4 Department of Biochemistry and Medical Genetics, University of Manitoba, Winnipeg, MB, Canada; 5 Department of Biochemistry, Western University, London, ON, Canada; 6 Department of Pediatrics and Child Health, University of Manitoba, Winnipeg, MB, Canada; 7 Department of Physiology and Pathophysiology, University of Manitoba, Winnipeg, MB, Canada

**Keywords:** Dentistry, Human genetics, Microbiome

## Abstract

Severe early childhood caries (S-ECC) is a multifactorial disease with strong evidence of genetic inheritance. Previous studies suggest that variants in taste genes are associated with dental caries due to the role of taste proteins in mediating taste preferences, oral innate immunity, and important host-microbial interactions. However, few taste genes have been investigated in caries studies. Therefore, the associations of genetic variants in sweet, bitter, umami, salt, sour, carbonation, and fat taste-related genes with S-ECC and plaque microbial composition (16S and ITS1 rRNA sequencing) were evaluated. The results showed that sixteen variants in seven taste genes (*SCNN1D, CA6, TAS2R3, OTOP1, TAS2R5, TAS2R60*, and *TAS2R4*) were associated with S-ECC. Twenty-one variants in twelve taste genes were correlated with relative abundances of bacteria or fungi. These results suggest that S-ECC risk and composition of the plaque microbiome can be partially influenced by genetic variants in genes related to taste sensation.

## Highlights

• Variants in taste-related genes may affect the oral bacteriome and mycobiome
• Taste-related genes may play a key role in dental caries development
• These studies may allow more personalized dental treatment plan for young children

## Graphical abstract

**Figure:**
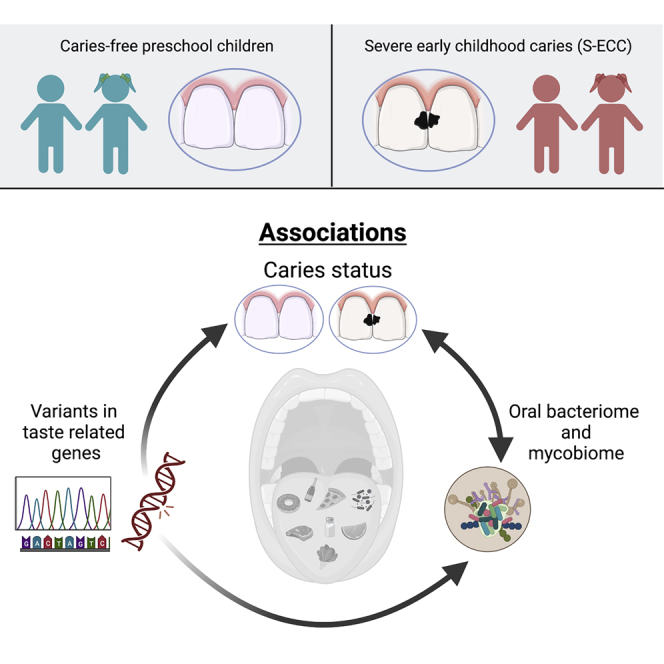

## Introduction

Early childhood caries (ECC), and its more severe form S-ECC, is a complex disease and many factors have been associated with its onset. These include diet, oral hygiene, enamel defects, oral microbiota, and saliva buffering capacity and flow rate.^1^^,^^2^ It has been estimated, based on twin studies, that the heritability of dental caries, or the proportion of variation that is due to genes, can reach over 50%.^3^^,^^4^

Genetics may influence multiple factors implicated in dental caries etiology. In recent years, evidence suggests that there is a plausible relationship between genetic variants in taste genes and enhanced dental caries risk or protection.^5^^,^^6^^,^^7^^,^^8^ Dietary choices are influenced by taste and studies have shown that children who are sweet likers tend to consume more sugar and be more prone to dental caries.^7^^,^^9^^,^^10^ Several studies have shown that genetic variants in sweet and umami taste receptors (*TAS1Rs* or T1Rs) and bitter taste receptors (*TAS2Rs* or T2Rs) affect individuals’ food preferences.^7^^,^^8^^,^^11^^,^^12^^,^^13^

Beyond taste sensation, taste receptors have been implicated in innate immune responses against oral bacteria.^14^^,^^15^^,^^16^ T2Rs are expressed in immune cells, such as macrophages, and are activated by bacterial molecules leading to increased intracellular Ca^2+^ and decreased cAMP levels. There is evidence that this Ca^2+^ signal stimulates the production of nitric oxide (NO) by epithelial cells, which besides having direct antibacterial and physiological effects, also enhances macrophage phagocytosis.^17^^,^^18^^,^^19^ The decrease in cAMP level after activation of T2Rs may also contribute to phagocytosis.^18^ In a study using knockout mice, it was shown that defects in taste signaling components can affect oral bacterial load and diversity.^20^

Taken together, these effects on taste preferences and innate immune responses mediated by taste-related proteins suggest that they may play a key role in dental caries development. Studies have already highlighted the link between *TAS2Rs* and *TAS1Rs*, sweet taste preference, high consumption of carbohydrates, and dental caries in children.^5^^,^^10^^,^^21^^,^^22^^,^^23^ However, to date, only a very limited number of taste genes have been investigated. Further, the role of salt, sour, fat, and carbonation taste sensing genes in dental caries development is not well characterized.

Salt taste sensation involves epithelial sodium channels (ENaCs α, β, γ, and δ) coded by *SCNN1A, SCNN1B, SCNN1G*, and *SCNN1D* genes.^24^ Sour (acidic) taste is mediated by H^+^-permeable channel otopetrin1 or OTOP1.^25^^,^^26^ The candidate fat taste receptors include the free fatty acid receptors FFAR1 (GPR40), FFAR2 (GPR43), FFAR3 (GPR41), and FFAR4 (GPR120).^27^ The taste of carbonation is encoded by carbonic anhydrase genes (*CA*s).^28^ Studies have shown that fatty acids that activate FFARs are associated with antimicrobial effects against *Streptococcus mutans* and *Candida albicans* oral infections.^29^^,^^30^ An association between the level of perception of salt taste and presence of dental caries lesions has also been previously suggested.^31^ Mutations in *CA* have been linked to the prevalence of dental caries in adolescents.^32^ A recent genome-wide association study identified a suggestive association between dental caries and a locus near *TAS2Rs* and a *CA*.^33^ Therefore, the role of bitter, sweet, umami, sour, salt, fat, and carbonation sensing taste genes in S-ECC and the oral microbial needs to be further investigated.

Given the strong evidence of a genetic contribution to dental caries susceptibility and the link among variants in taste genes, taste preferences, and oral innate immunity, it was hypothesized that variants in taste genes are associated with S-ECC and may partially explain the differences between the dental plaque microbiome of caries-free children and those with S-ECC. To test this hypothesis, the interplay between variants (single nucleotide polymorphisms or SNPs and short insertions and deletions or INDELs) in genes associated with taste (sweet, umami, bitter, salt, sour, carbonation, and fat), the plaque microbiome, and susceptibility to S-ECC was evaluated.

## Results

### Demographic characteristics of the study participants

**Figure 1:**
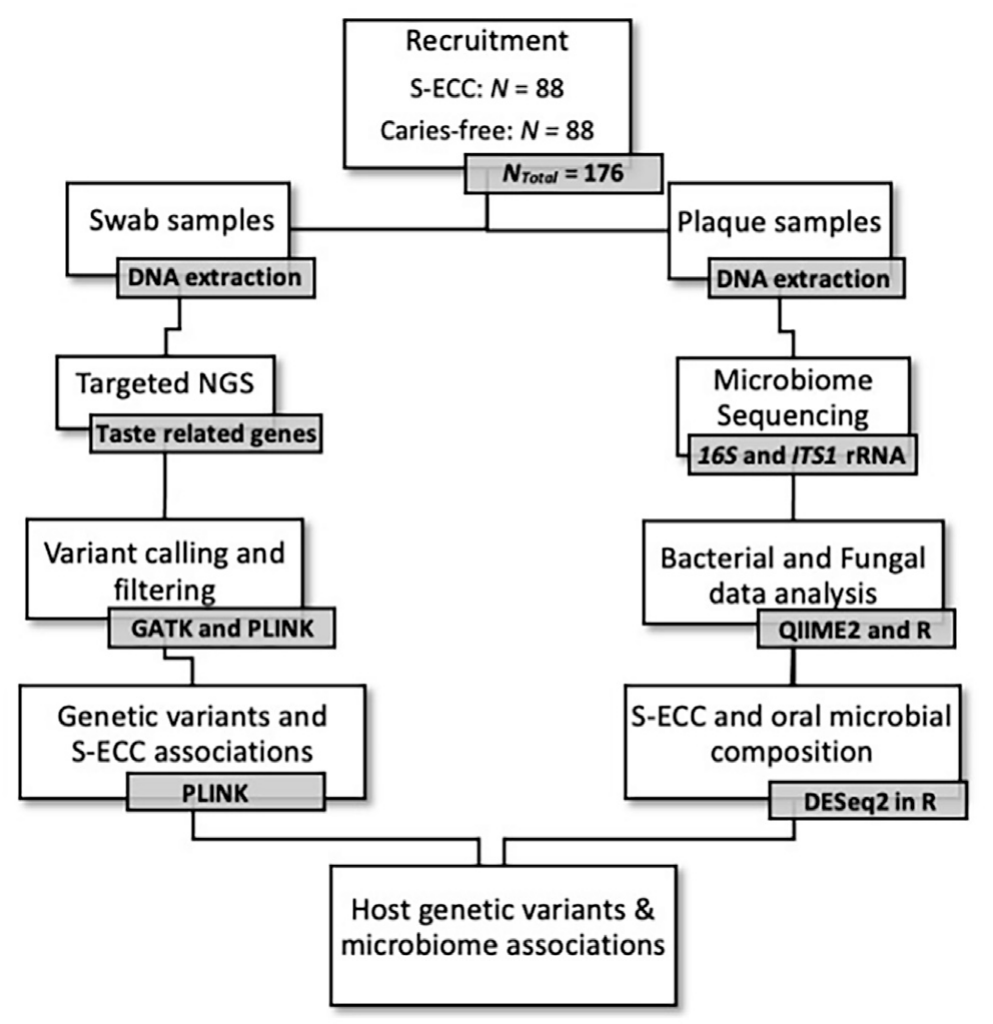
Flowchart of the study design

The characteristics of the study participants are shown in Table 1. There was no difference between the caries-free and S-ECC groups with respect to age or sex. A total of 98.3% of the parents or legal caregivers self-reported that their children had good or very good overall health and one parent/caregiver from each group reported that their child had fair overall health. Majority of the children were not taking any medication, except for four caries-free children that were taking medication for asthma or hypothyroidism or acetaminophen. The study design is shown in Figure 1.

**Table 1:** Characteristics of study population

	Caries-free (*N* = 88)	S-ECC (*N* = 88)	*p*
Age (months, mean ± SD)	45.3 ± 14.7	44.9 ± 11.8	0.82

**Sex**			

Female (n (%), *N* = 94)	43 (48.9%)	51 (58%)	0.23
Male (n (%), *N* = 82)	45 (51.1%)	37 (42%)	

p-value derived from Student T test or χ^2^ test. S-ECC, severe early childhood caries.

### Association between taste genetic variants and S-ECC status

A total of 215 variants and 176 samples passed quality control (See Figure S1). The case-control allelic association analysis showed that 16 variants in seven genes are significantly associated with S-ECC (adj. *p* < 0.05, Table 2). Logistic regression analysis under an additive genetic model including sex and age as covariates showed that 15 of these 16 variants remained associated with S-ECC risk or protection (adj. *p* < 0.05, Table 3). An additional *CA6* variant, rs2274330, was also identified as significant (Table 3).

**Table 2:** Allelic association of genetic variants with severe early childhood caries (S-ECC), ranked by adjusted p value and gene names

Gene	Variants	Location (GRCh38)	Effect allele	Frequency of effect allele		χ^2^	*p*	OR	*Adj.p*^a^	Type of variant
				Case (S-ECC)	Controls (Caries-free)					
*SCNN1D*	rs111819661	chr1: 1287578	T	0.29	0.02	50.4	1.26E-12	23.53	<0.001	Missense p.Arg461Cys
	rs2273275	chr1: 1290841	A	0.31	0.60	29.77	4.86E-08	0.30	<0.001	Intronic
	rs13306638	chr1: 1290851	A	0.31	0.09	25.75	3.89E-07	4.43	<0.001	Intronic
	rs1570867	chr1: 1290579	C	0.34	0.61	25.66	4.06E-07	0.33	<0.001	Intronic
	rs609805	chr1: 1291509	A	0.35	0.16	16.38	5.19E-05	2.80	0.0112	Missense p.Gly770Arg
	rs910556	chr1: 1287315	C	0.42	0.22	15.97	6.45E-05	2.55	0.0139	Intronic
	rs586965	chr1: 1290968	G	0.56	0.35	15.67	7.54E-05	2.36	0.0162	Intronic
*TAS2R60*	rs35195910	chr7: 143443837	G	0.20	0.03	22.86	1.74E-06	6.95	0.0004	Disruptive inframe deletion 392_394delTCT p.Phe131del
*TAS2R5*	rs2227264	chr7: 141790438	G	0.23	0.46	21.42	3.68E-06	0.34	0.0008	Missense p.Ser26Ile
*TAS2R3*	rs2270009	chr7: 141764965	C	0.25	0.49	21.39	3.75E-06	0.35	0.0008	Synonymous
	rs765007	chr7: 141764114	T	0.25	0.46	16.57	4.69E-05	0.39	0.0101	5′-UTR
*OTOP1*	rs145781170	chr4: 4197180	A	0.14	0.01	20.1	7.35E-06	13.74	0.0016	Missense p.Leu552Phe
	rs17697262	chr4: 4198033	A	0.29	0.11	18.26	1.93E-05	3.37	0.0041	Synonymous
*TAS2R4*	rs2234001	chr7: 141778774	G	0.25	0.47	18.59	1.62E-05	0.37	0.0035	Missense p.Val96Leu
	rs2234002	chr7: 141779000	G	0.25	0.47	18.3	1.89E-05	0.38	0.0041	Missense p.Ser171Asn
*CA6*	rs2274329	chr1: 8949392	C	0.04	0.17	15.98	6.41E-05	0.20	0.0138	Missense p.Gly70Ala

a Adjusted *P* (corrected for multiple testing by Bonferroni adjustment test) less than 0.05 were considered statistically significant. Only significant associations are shown. χ^2^, basic allelic test Chi-square. The basic allelic test compares frequencies of alleles in cases versus controls. OR, odds ratio. 5′-UTR, 5′ untranslated region.

**Table 3:** Results of logistic regression model showing the association of genetic variants with severe early childhood caries (S-ECC), ranked by adjusted p value and gene names.

Gene	Variant	Location (GRCh38)	Allele	MAF	OR	95% CI	*p*	*Adj.p∗*
*SCNN1D*	rs111819661	chr1:1287578	T	0.15	35.06	10.22–120.3	<0.001	<0.001
	rs2273275	chr1:1290841	A	0.46	0.30	0.1895–0.4898	<0.001	0.0002
	rs13306638	chr1:1290851	A	0.2	4.82	2.5–9.29	<0.001	0.0005
	rs1570867	chr1:1290579	C	0.47	0.32	0.1967–0.5195	<0.001	0.001
	rs910556	chr1:1287315	C	0.32	3.25	1.877–5.638	<0.001	0.005
	rs586965	chr1:1290968	G	0.46	2.87	1.713–4.822	<0.001	0.013
	rs609805	chr1:1291509	A	0.25	3.12	1.769–5.498	<0.001	0.017
*CA6*	rs2274329	chr1:8949392	C	0.10	0.15	0.06031–0.3735	<0.001	0.009
	rs2274330	chr1:8949411	C	0.11	0.21	0.09–0.475	<0.001	0.041
*TAS2R3*	rs2270009	chr7:141764965	C	0.37	0.34	0.2053–0.5493	<0.001	0.003
	rs765007	chr7:141764114	T	0.36	0.36	0.2148–0.5928	<0.001	0.014
*OTOP1*	rs17697262	chr4:4198033	A	0.2	4.18	2.178–8.011	<0.001	0.003
*TAS2R5*	rs2227264	chr7:141790438	G	0.34	0.33	0.202–0.5537	<0.001	0.004
*TAS2R60*	rs35195910	chr7:143443837	G	0.12	6.88	2.727–17.34	<0.001	0.009
*TAS2R4*	rs2234002	chr7:141779000	G	0.36	0.36	0.221–0.5892	<0.001	0.009
	rs2234001	chr7:141778774	G	0.36	0.38	0.2369–0.6136	<0.001	0.014

Chr, chromosome. MAF, minor allele frequency. 95% CI, 95% confidence interval. OR, odds ratio. ∗Corrected for multiple testing by Bonferroni adjustment test. Adjusted p < 0.05 were considered statistically significant. Only significant associations are shown.

**Figure 2:**
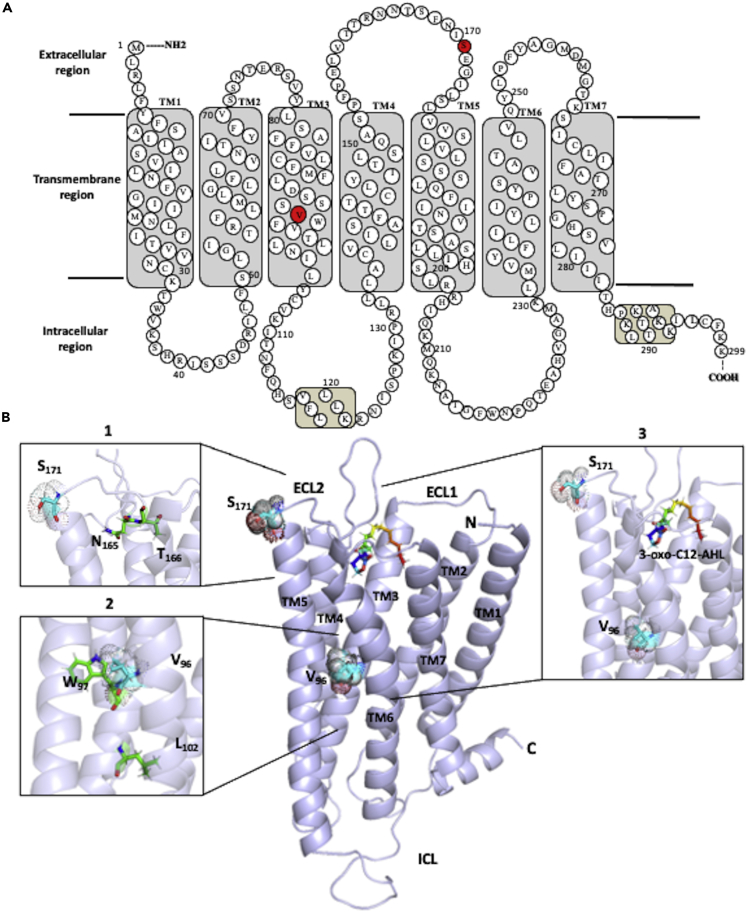
2D and 3D representations of T2R4 showing the predicted location of V96 and S171 (A) 2D representation of the amino acid sequence of T2R4 containing 299 residues. T2R4 forms a short N terminus, 7 transmembrane (TM) helices, three extracellular loops (ECLs), three intracellular loops (ICLs), and an intracellular C terminus. The position of the amino acid affected by the S-ECC-associated SNPs is highlighted in red. V96 is present in the TM3 and S171 is located in ECL2. (B) Three-dimensional model of T2R4 docked with a quorum sensing molecule (3-oxo-C12-AHL) as described in Jaggupilli et al. (2018). [1] The S171 present in ECL2 is in close proximity to amino acids (N165 and T166) previously shown to be important for T2R4 ligand binding.(Refs.^34^^,^^35^) [2] The V96 present in the central or core region of TM3 is next to the highly conserved amino acids W97 and L102. Previously, this region was shown to play an important structural role (helix packing) in T2R1.(Ref.^62^) [3] Side view of the ligand binding pocket of T2R4 bound to 3-oxo-C12-AHL. The T2R4 models are from previous studies.(Ref.^34^).

To understand how the genetic variants might be influencing protein structure and function, the bitter taste receptor T2R4 was analyzed as a test case. Two loci in the gene encoding T2R4 were significantly associated with S-ECC (Table 3). T2R4 has been previously characterized by site-directed mutational studies from our group.^34^^,^^35^ Therefore, the T2R4 molecular model was used from these studies to visualize the predicted location of the amino acids that are affected by the two S-ECC-associated variants in *TAS2R4* (rs2234001 and rs2234002). The location of V96 is on the transmembrane helix 3 (TM3) and S171 is present in the extracellular loop 2 (ECL2) (Figure 2).

### Association between bacterial and fungal microbiota and S-ECC status

**Figure 3:**
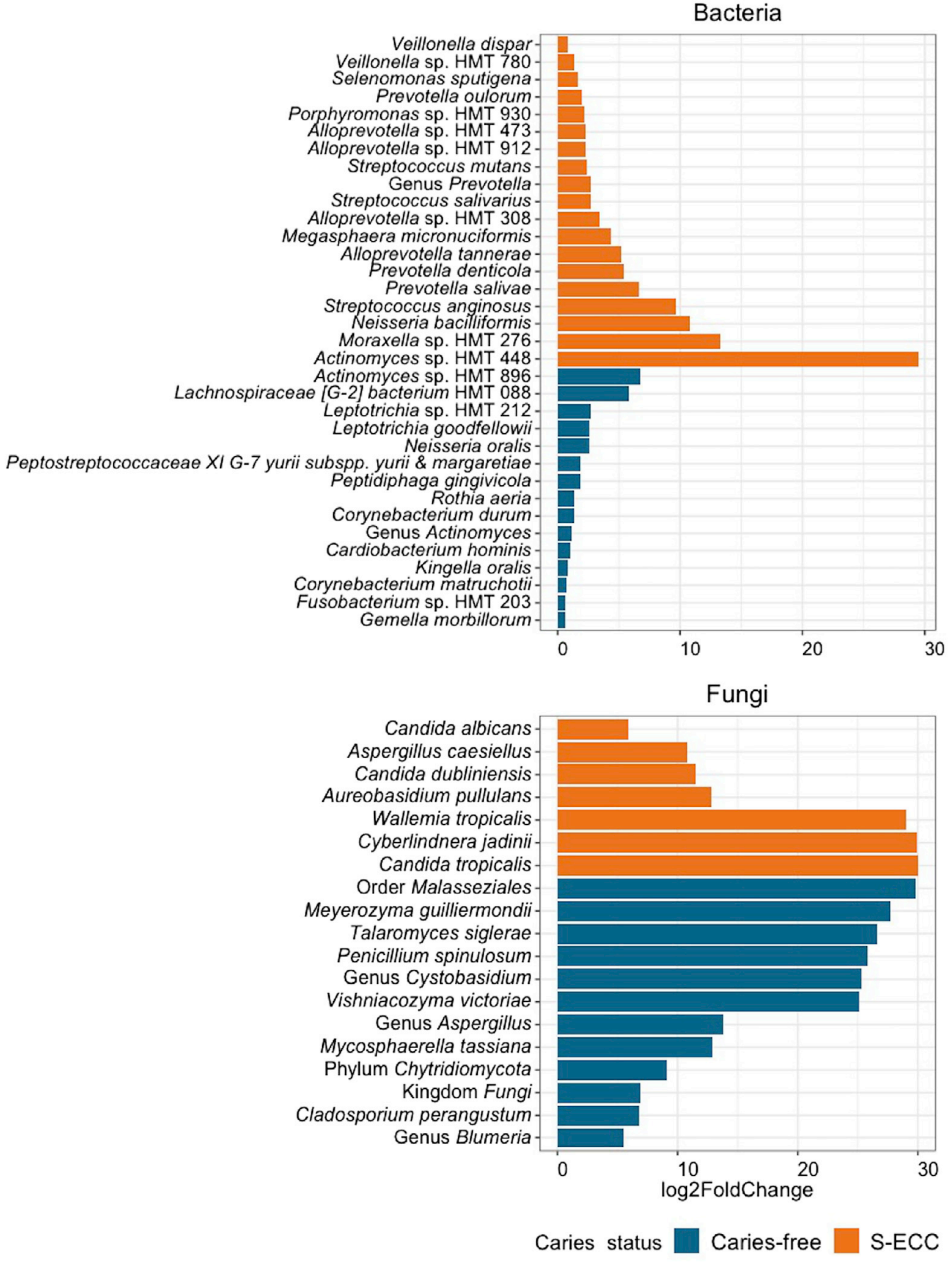
Differential abundance analysis of the S-ECC and caries-free groups using DESeq2 negative binomial Wald test The plots show the relative fold change in the abundance of bacterial and fungal taxa that were significantly more abundant in the caries-free (blue) or in the S-ECC (orange) groups. All species listed have an FDR adjusted *p* < 0.05. S-ECC, severe early childhood caries.

The 16S and ITS rRNA gene sequencing generated an average of 69,243.47 (16S) and 76,838.11 (ITS) reads for the S-ECC group and 70,640.77 (16S) and 60,032.80 (ITS) reads for the caries-free groups. Nine phyla and at least 61 genera of bacteria were identified with *Neisseria*, *Actinomyces*, *Streptococcus*, and *Veillonella* being the most abundant. At least 161 bacterial species were identified. DESeq2 differential abundance analysis identified 34 bacterial taxa that were significantly enriched or depleted in the supragingival plaque of children with S-ECC or caries-free controls (Figure 3). For ITS1 rRNA sequences, 10.8% of the samples did not pass quality control or had low number of reads and were removed from analyses that included fungal data. Overall, at least 67 genera and 79 fungal species were found in at least two samples. *Ascomycota* and *Basidiomycota* were the most abundant phyla and *Candida* was the most abundant genus identified. The abundance of 19 fungal taxa, including three *Candida* species, was significantly different between the caries-free and S-ECC groups (Figure 3). In previous studies, the oral microbiome of children with S-ECC and caries-free controls was characterized with more detailed analyses, including machine learning.^36^^,^^37^

### Associations between host genetic variants and the abundance of oral microbes

**Figure 4:**
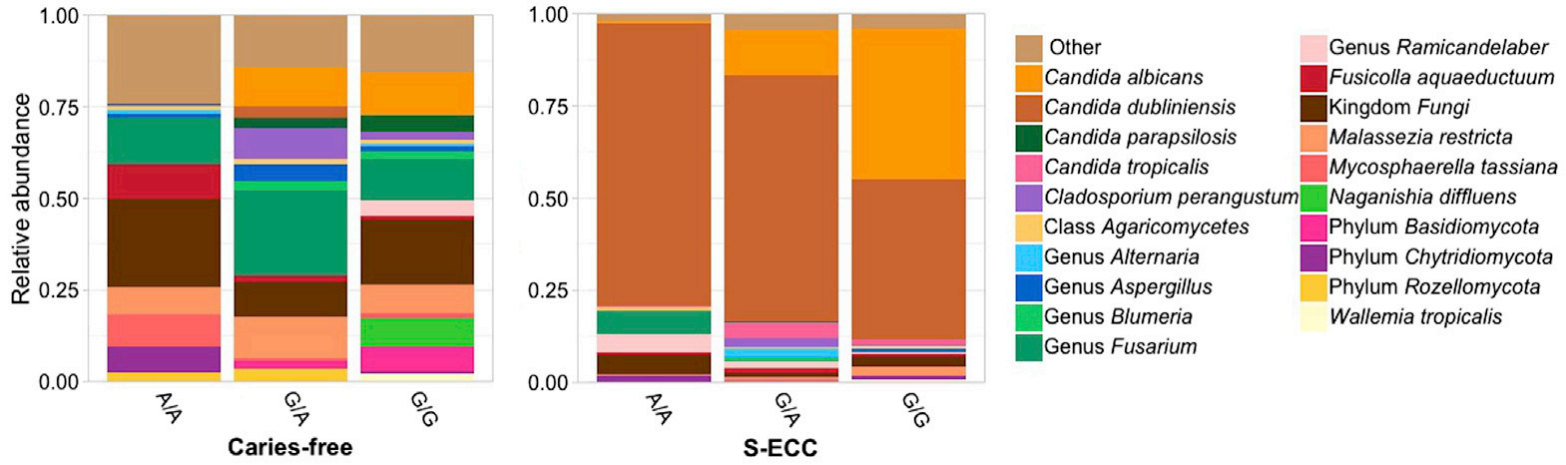
Relative abundance of the top 20 most abundant fungi according to S-ECC status and *TAS2R38* rs1726866 genotypes (A/A, G/A, G/G)

**Figure 5:**
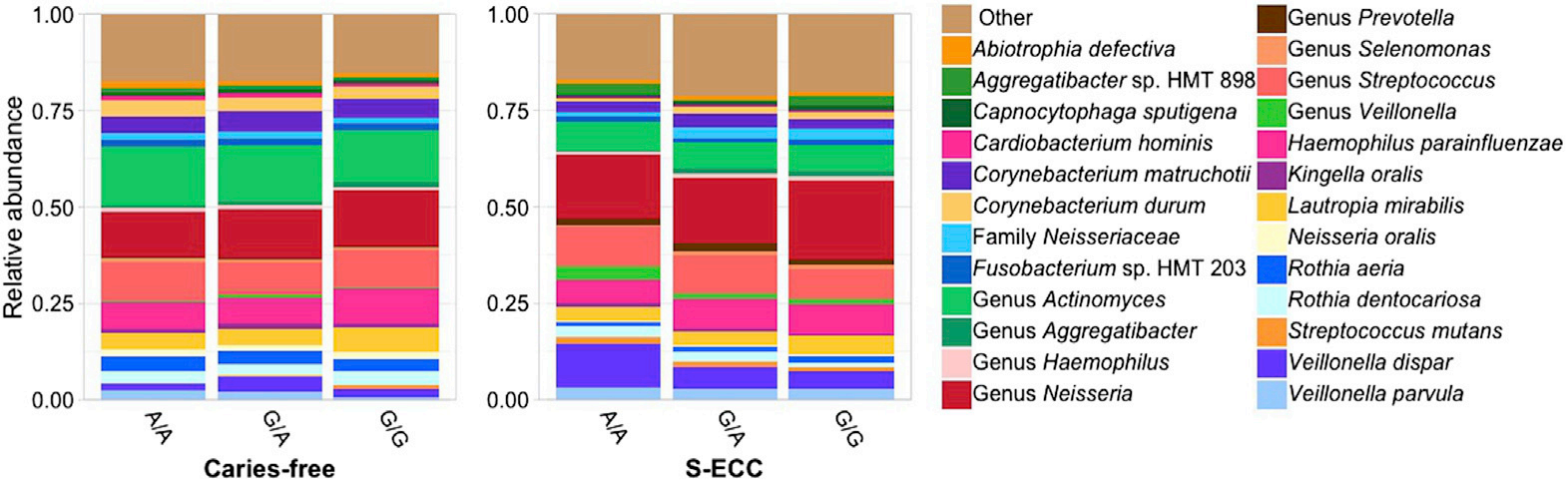
Relative abundance of the top 25 most abundant bacteria according to S-ECC status and *TAS2R38* rs1726866 genotypes (A/A, G/A, G/G)

Significant associations among 21 host variants in salt, bitter, sweet, umami, fat, and sour genes and the relative abundances of 16 bacterial or fungal taxa were identified (Table 4). No significant associations were observed between bacterial or fungal alpha diversity and genetic variants (Adj. *p* > 0.05). The strongest association was with rs63982 located in the *SCNN1B* gene, which negatively correlated with *Neisseria* sp. (Adj. p = 0.005, Table 4). The second strongest association was on *SCNN1D* rs111819661, which was also associated with increased odds of S-ECC (Table 3). The minor allele T of rs111819661 was negatively correlated with an unclassified *Actinomyces* species (Adj. p = 0.006, Table 4). Interestingly, *TAS2R38* rs1726866 was associated with *Candida albicans*, which has been implicated in dental caries development. To further explore this association, the relative abundance of the most abundant fungal and bacterial taxa according to S-ECC status and rs1726866 genotypes was investigated and is shown in Figures 4 and 5. Children with S-ECC that have the A/A genotype had decreased relative abundance of *C. albicans* (Figure 4). However, the relative abundance of *C. dubliniensis* in that group was increased compared to the G/G genotype. The opposite pattern was observed in children with the G/G genotype, who also showed lower relative abundances of the S-ECC-associated bacteria *S. mutans*, *Veillonella parvula,* and *V. dispar* (Figure 5). Other significant associations among taste gene variants and oral microbial composition are shown in Table 4.

**Table 4:** Association between host genetic variants and oral bacterial and fungal taxa.

Gene	Genetic variants	Location	Effect allele	Beta	Adj. *p*	Taxa
**BACTERIA (*N* = 176)**						

*SCNN1B*	rs63982	chr16:23352760	C	−0.464	0.005	Genus *Neisseria*
	c.949A>G	chr16:23371367	G	−1.818	0.011	*Cardiobacterium hominis*
	rs238547	chr16:23348878	C	0.855	0.015	*Prevotella oris*
	c.701A>G	chr16:23355414	G	−2.191	0.028	*Corynebacterium matruchotii*
*SCNN1D*	rs111819661	chr1:1287578	T	−0.504	0.006	Genus *Actinomyces*
	rs770027731	chr1:1287957	C	6.913	0.036	*Kingdom bacteria*
	rs910556	chr1:1287315	C	−0.729	0.045	*Neisseria oralis*
*CA6*	rs3737665	chr1:8970905	T	−0.777	0.012	Genus *Capnocytophaga*
	rs2274330	chr1:8949411	C	1.815	0.036	*Prevotella salivae*
	rs2274331	chr1:8949488	A	1.815	0.036	*Prevotella salivae*
*FFAR3*	c.341T>C	chr19:35359231	C	3.593	0.014	*Veillonella* sp. HMT 780
	rs4806132	chr19:35358981	G	−0.452	0.033	Genus *Actinomyces*
*TAS1R3*	c.1600 + 27delG	chr1:1333401	C	−2.085	0.014	*Cardiobacterium hominis*
*SCNN1G*	rs13306654	chr16:23213196	G	0.414	0.019	*Bergeyella* sp. HMT 322
	rs13306653	chr16:23213095	A	0.391	0.048	*Bergeyella* sp. HMT 322
*TAS2R3*	rs2270009	chr7:141764965	C	−0.673	0.038	*Bergeyella* sp. HMT 907
*CA7*	c.453 + 11delG	chr16:66851567	T	−1.428	0.046	*Porphyromonas paster*

**FUNGI (*N* = 157)**						

*OTOP1*	rs145781170	chr4:4197180	A	−4.334	0.011	Genus *Blumeria*
*TAS2R38*	rs1726866	chr7:141972905	A	−2.623	0.026	*Candida albicans*
*TAS2R41*	rs1404635	chr7:143478061	A	−1.912	0.040	*Candida dubliniensis*
*TAS1R1*	rs34160967	chr1:6575246	A	−4.708	0.047	*Candida dubliniensis*

Beta: regression coefficient. Adj. *p* < 0.05 are corrected for multiple testing by Bonferroni adjustment test.

Del: deletion.

## Discussion

Findings from this study reveal that genetic variants in taste-related genes are associated with S-ECC risk or resistance as well as with the abundance of oral bacteria and fungi. The influence of some of the taste-related genes in dental caries risk or protection has been proposed. Many studies have reported a link between taste receptor gene polymorphisms and dental caries risk.^5^^,^^6^^,^^22^^,^^38^^,^^39^ However, the majority of these studies focused only on *TAS2R38* and/or *TAS1R2/3*. In this study, genes involved in basic taste modalities, carbonation, and candidate fat receptors were assessed. Among them, at least one gene representing each of the taste modalities was associated with dental caries and/or the composition of the oral microbiome.

SNPs in *TAS2Rs* have been associated with variability in taste preferences and innate immune responses to oral and systemic infections. Previous studies evaluating the role of taste receptors in dental caries risk/protection have identified *TAS2R38* as a key player.^5^^,^^6^ Here, no significant associations between *TAS2R38* and dental caries in children were identified, which supports the findings from a recent cross-sectional study (*N* = 65).^39^ However, a negative correlation between *TAS2R38* rs1726866 and *Candida albicans* (Adj. *p* = 0.026) was observed. Intriguingly, the rs1726866 (A) allele represents the “non-taster” phenotype that has been previously associated with increased risk of dental caries and *Streptococcus mutans* levels.^5^^,^^8^ The differences in the relative abundances of *C. albicans* and *C. dubliniensis* that were observed between children with the A/A (non-taster) genotype and children with the G/G (“supertaster”) genotype suggest that *TAS2R38* rs1726866 genotype may influence the composition of the oral mycobiome due to their role in modulating oral innate immune responses and/or taste preferences, which can influence the children’s diet.^5^^,^^14^^,^^23^ However, whether these findings have clinical significance must be further investigated. Ultimately, children with S-ECC had higher abundance of *Candida* spp. compared to caries-free controls, regardless of their genotype, highlighting the importance of this yeast in dental caries development. Previous studies from our group showed that *C. dubliniensis* is strongly associated with S-ECC.^36^^,^^37^ The presence of deep cavities in children with S-ECC provides a unique environment for the growth of certain microbial species which may contribute to changes in their plaque microbial profile.^40^ Therefore, future studies should focus on determining what the role of *C. dubliniensis* in caries development is.

Interestingly, findings from this study point to a protective effect of *TAS2R4* variants against dental caries. This agrees with a previous study which showed that *TAS2R4*-rs2234001 G allele was associated with lower caries experience in adults.^39^ T2R4 is a well-characterized member of the T2R family. It is widely distributed in oral tissues and is expressed in immune cells (Gopallawa et al., 2020; Xi et al., 2022). Furthermore, previous studies from our group showed that T2R4 can be activated by multiple quorum sensing molecules and antibiotics.^34^^,^^35^ The amino acids that are affected by *TAS2R4* rs2234001 and rs2234002 are present in the TM3 and ECL2, respectively (Figure 2). Intriguingly, previous studies showed that mutations in ECL2 can lead to loss of function by affecting the interaction and activation of T2R4 by its ligands.^34^^,^^35^ Although the effect of S171N and V96L substitutions in T2R4 was not previously investigated, it can be hypothesized based on these previous findings that S171N may affect the interaction of T2R4 with certain ligands.^34^^,^^35^ The region where V96 is located may be crucial for helix packing and maintenance of the protein structure. Therefore, V96L may have a subtle influence on protein structure and function. Site-directed mutational studies should be performed to test these hypotheses.

Collectively, findings from this study and others suggest a critical role of T2R4 in host-microbial interactions, innate immune modulation, and regulation of the mechanism of action of drugs. The potential role of T2R4 in dental caries protection has an exciting therapeutic or pharmacological application as studies have shown that T2R4 function or expression can be modulated with known agonists (e.g., the quinine’s chaperone activity) and blockers.^41^^,^^42^^,^^43^ Hence, there is an urgent need for the characterization of the molecular mechanisms involved in T2R4 protective effect on S-ECC and the development of effective prevention strategies incorporating this receptor.

Three other *TAS2Rs* showed associations with S-ECC. *TAS2R5*-rs2227264, and *TAS2R3*-rs2270009 and -rs765007 were associated with decreased odds of S-ECC, while opposite effect was observed with *TAS2R60*-rs35195910. *TAS2R3*, -5, and -60 are also expressed in multiple oral cells.^19^ To our knowledge, this is the first study to suggest an association between *TAS2R5* and *TAS2R60* variants in S-ECC risk or protection. A recent genome-wide association study ( *N* = 3,686) including a multiethnic cohort identified a suggestive association between dental caries in permanent dentition and a locus near *TAS2R38, TAS2R3, TAS2R4,* and *TASR25* on chromosome 7.^33^ They also identified possible associations with a locus near *CA9* and dental caries in primary dentition. They suggested that susceptibility to dental caries in different dentition may be influenced by different genetic loci, highlighting the importance of more genetic studies in preschool children to determine the proportion of ECC susceptibility that can be attributed to genetic factors.

Other taste receptors and channels may also be implicated in caries susceptibility due to their roles in taste modulation or other physiological effects. For instance, studies have suggested that FFAR2 and FFAR3 activated by short-chain fatty acids (SCFAs) in the gut may play a role in cytokine secretion and recruitment of immune cells.^44^^,^^45^ However, the role and level of expression of *FFARs* in the oral cavity is poorly characterized. A recent study showed, using qRT-PCR and western blotting, that *FFAR4* and *FFAR2* are expressed in the human fungiform papillae and that the level of expression of *FFAR2* was linked to dietary fat intake.^46^ A recent study showed that FFAR3 is functionally expressed in human airway smooth muscle and can induce increase in intracellular Ca^2+^ levels via the Gαi/βγ- PLCβ-IP_3_ pathway once activated by SCFAs.^47^ Their findings also suggest an association between FFAR3 and detrimental effects on airway smooth muscle tone. FFAR2 and FFAR3 have also been associated with inflammation and metabolic diseases such as diabetes and obesity.^48^^,^^49^^,^^50^ In the current study, although the association between *FFAR3* variants and S-ECC susceptibility did not achieve significance after correction for multiple testing, significant correlations between *FFAR3* variants and the abundance of oral bacterial species were observed. Therefore, the role of SCFAs and FFARs in dental caries development must be further investigated.

The role of salt and sour ion channels in dental caries has been poorly characterized. This study found strong associations between variants in sour and salt taste genes, particularly *SCNN1D,* and S-ECC risk or protection. A recent study evaluated the association between sweet, salt, sour, and bitter taste perception and dental caries experience in children (*N* = 200, 6–12 years old).^31^ They found significant correlations between the level of perception of sweet and salt tastes and occlusal dental caries lesions. Results from this study suggest that the link between salt and sour taste variability and dental caries risk/protection may be related to the role of *OTOP1* and *SCNN1s* variants in influencing the composition of the oral microbiome and this should be further explored in future studies.

In this study, genetic variants in *CAs*, which are highly expressed in ameloblasts and may be essential for tooth formation by providing local buffering, were also assessed. In fact, mutations in *CA* genes have been linked to dental defects.^51^ CA6 (gustin) and CA7 are both expressed in salivary glands and ameloblasts, and have been linked to Sjogren’s syndrome.^52^^,^^53^^,^^54^ Intriguingly, CA6 has been shown to play a role in dental enamel formation and bitter taste sensation. It is also present in saliva and breast milk.^53^^,^^55^^,^^56^^,^^57^ Furthermore, the association between *CA6* and dental caries has been previously investigated. A significant link between *CA6* rs10864376, rs3737665, rs12138897 CCC, and TTG haplotypes with low and high prevalence of dental caries and *S. mutans* in Swedish adolescents was reported.^32^ That study also found an association between *CA6* polymorphisms and dental plaque microbial composition. In contrast, another study found no association between *CA6* polymorphisms and dental caries in Brazilian children.^58^ Nonetheless, they identified a positive association between *CA6*-rs2274327 and salivary buffer capacity, with the T allele being more frequent in children with lower buffer capacity.^58^ Similar results were observed in Turkish adults.^6^ The present study showed that *CA6* rs2274330 and rs2274329 were associated with decreased odds of having S-ECC. Moreover, one *CA7* small deletion and three *CA6* SNPs (rs2274330, rs2274331, and rs3737665) significantly associated with the abundance of oral bacterial species. Taken together, results from this study and findings from the literature suggest that *CA* polymorphisms may influence dental caries susceptibility, which could be related to tooth defects, taste preferences, oral microbial composition, or salivary properties, deserving further investigation.

In this study, associations between S-ECC and intronic and synonymous variants were identified. Five variants in *SCNN1D* (rs2273275, rs13306638, rs1570867, rs910556, and rs586965) located on chromosome 1 were in intronic regions. One variant in *TAS2R3* (rs2270009), one in *OTOP1* (rs2270009), and one in *CA6* (rs2274330) on chromosomes 7, 4, and 1, respectively, were synonymous. Although silent mutations do not alter the protein sequence, they may have an effect in its function by affecting mRNA splicing, speed of translation, protein folding, and posttranslational modifications.^59^ Similarly, intronic variants can impact protein function, especially if they are near to splice sites, which are important for pre-mRNA splicing, i.e. removal of intronic (non-coding) sequences. Diseases have been associated with splicing defects that result in exon skipping or intron retention, due to intronic variants.^60^^,^^61^

In conclusion, in this study, the interplay among the dental plaque microbiome, variants in genes associated with taste, and S-ECC was evaluated. The results showed that several genetic loci were associated with S-ECC. Significant associations between genetic variants and the relative abundance of bacterial and fungal taxa were also identified. These results suggest that genetic variants of taste-associated genes may be key factors in dental caries susceptibility. These results could guide further multidisciplinary studies with the goal to create better tools for determining S-ECC risk, which may allow more personalized dental diagnosis, treatment, and prognosis for young children.

### Limitations of the study

This is the first study to take a comprehensive approach and analyze genetic variants of multiple taste-related genes and their association with the oral bacteriome and mycobiome in S-ECC. However, its limitations include the lack of information about some potentially confounding variables, e.g., socioeconomic status (SES) and lifestyle, that could have contributed to the microbial associations observed in this study. The study has a small sample size, but it was still possible to identify significant associations. All procedures were done under aseptic conditions to avoid contamination; however, no negative or positive controls were used for the microbiota DNA extraction or data analysis. It is also important to note that at least one variant from each selected genes passed quality control analyses, except for some *TAS2Rs*. Among *TAS2Rs*, only variants from *TAS2R*-1, -3, -4, -5, -16, -38, -40, -41, and -60 passed quality control and were analyzed. This may be due to the stringent filters that were applied to make sure that only high-quality reads and variants were evaluated. The downstream taste signaling associated genes that were not explored in this study need to be evaluated in future studies.

## STAR★Methods

### Key resources table

**Table:** 

REAGENT or RESOURCE	SOURCE	IDENTIFIER
**Critical commercial assays**		

QIAamp DNA Mini Kit	Qiagen	Cat # 51306
DNeasy PowerSoil Kit	Qiagen	Cat # 12888-100

**Deposited data**		

16S and ITS1 rRNA gene sequences	This paper	PRJNA555320

**Software and algorithms**		

QIIME2 version v.2018.11	(Bolyen et al., 2019)	https://docs.qiime2.org/2018.11/tutorials/
R version v.3.6.2	(R Core Team, 2019)	https://www.R-project.org/
qiime2R package v.0.99.13	(Bisanz, 2018)	https://github.com/jbisanz/qiime2R
microbial package v.0.0.19	(Guo and Gao, 2021)	https://CRAN.R-project.org/package=microbial
DESeq2 package v.1.26.0	(Love et al., 2014)	https://bioconductor.org/packages/release/bioc/html/DESeq2.html
Phyloseq package v.1.30.0	(McMurdie and Holmes, 2013)	https://joey711.github.io/phyloseq/
Genome Analysis Toolkit (GATK) v.4.2.0 best practices pipeline	(Van der Auwera et al., 2020)	https://gatk.broadinstitute.org/hc/en-us/categories/360002302312
PLINK version v.1.9	(Purcell et al., 2007)	https://zzz.bwh.harvard.edu/plink/data.shtml#plink
SnpEff v.5.0e	(Cingolani et al., 2012)	https://pcingola.github.io/SnpEff/se_running/

### Resource availability

#### Lead contact

Further information should be directed to Dr. Prashen Chelikani (Prashen.Chelikani@ umanitoba.ca).

#### Materials availability

This study did not generate new unique reagents.

### Experimental model and subject details

#### Study population and design

One hundred and seventy-six children younger than 72 months of age were recruited between 2017 and 2020 in Winnipeg-MB, Canada. In a previous study, we reported sex-specific differences in the oral microbiome of children with S-ECC.^36^ Therefore, male and female children were included in this study, and sex was considered during the statistical analysis. For this cross-sectional study, eighty-eight children with severe early childhood caries were recruited at the day of their scheduled dental rehabilitative surgery under general anesthesia at the Misericordia Health Center. The caries-free children (*N* = 88) were recruited from the community and were included in the study after a clinical examination by a dentist confirmed their caries-free status (dmft index = 0, i.e., no decayed, missing, or filled primary tooth surface; no incipient lesions). Inclusion criteria: children less than 72 months of age who were caries-free (dmft = 0) or have been diagnosed with S-ECC (based on the American Academy of Pediatric Dentistry definition).^63^ Exclusion criteria: children older than 72 months of age, current use of antibiotics, and children who did not satisfy the case definition of S-ECC.

The parents or legal caregivers provided written informed consent and the study protocol was approved by the University of Manitoba’s Health Research Ethics Board (HREB HS23754-H2020:150) and the Misericordia Health Centre. Figure 1 shows an overview of the overall study design.

### Method details

#### Sample collection and DNA extraction

The supragingival plaque sample was collected from all available tooth surfaces with a sterile interdental brush. The oral swab sample was collected with a sterile polyester-tipped applicator (Fisher Scientific) by swabbing the buccal mucosa and anterior floor of the mouth under the tongue. The dental plaque and oral swabs were stored in 1mL of RNAprotect Reagents (Qiagen, Hilden, Germany) and stored at −80°C until further analysis.

Total DNA was extracted from oral swabs and dental plaque samples using QIAamp DNA mini kit (Qiagen) or DNeasy PowerSoil Kit (Qiagen) following manufacturer`s protocol. An additional enzymatic digestion step with lysozyme treatment (20 μg/ml lysozyme in a buffer containing 20 mM Tris HCl, pH 8; 1.2% Triton X 100; and 2mM EDTA, at 37°C for 30 minutes) was performed before DNA extraction with QIAamp DNA mini kit.

#### *16S* and *ITS* rRNA gene sequencing and data analysis

The DNA extracted from supragingival plaque was used for microbiome sequencing as described previously.^36^^,^^37^ The library preparation and sequencing (MiSeq PE250, Illumina Inc., San Diego, CA, USA) were performed by Génome Québec Innovation Center (Montréal, Canada). The paired-end reads were analyzed using QIIME2 v.2018.11 pipeline.^64^ DADA2 was used for quality trimming, filtering, and merging of sequences, resulting in the amplicon sequence variant (ASVs) tables (Callahan et al. 2016^65^). ITS1 sequences were trimmed before using the Q2-ITSxpress QIIME2 plugin, with default parameters (Rivers et al. 2018^66^). Taxonomic assignment was performed using sklearn against the HOMD (version 15.1) and UNITE (version 8.2; QIIME developer release) databases for bacteria and fungi, respectively.^67^^,^^68^^,^^69^ The data was imported into R v.3.6.2^70^ using the qiime2R package (version 0.99.13).^71^ The downstream analyses were performed using phyloseq package (version 1.30.0) in R.^72^ ASVs present only in one sample were removed. The relative abundance of the top bacterial and fungal taxa were visualized using the microbial package (version 0.0.19).^73^

#### Targeted sequencing of candidate genes

The DNA extracted from buccal swab was used for sequencing of the taste genes. The targeted sequencing of 39 genes (see Table S1) was performed by Génome Québec Innovation Center (Montréal, Canada). The primers used are listed in Table S2. Paired-end sequencing was performed on the NovaSeq6000 SP PE250 (Illumina Inc., San Diego, CA, USA) platform after library preparation was carried out with Fluidigm Access Array technology (Fluidigm, South San Francisco, CA, USA).

The sequence data were processed using the Genome Analysis Toolkit (GATK v.4.2.0, Broad Institute) best practices pipeline.^74^ A total of 2,095 genetic variants (SNPs and INDELS) were called. Additional filtering and quality control was performed in PLINK (v.1.9).^75^^,^^76^ Variants with significantly different (p < 0.00001) missing data rate between cases and controls, > 5% missing genotype call rate, minor allele frequency (MAF) < 0.01, mean sequencing depth <10x, and Hardy–Weinberg equilibrium (HWE) p < 0.05 in controls were removed. Samples with a genotype failure rate >0.2 were also removed. All samples and 215 variants passed quality control. The filtered variants were annotated using SnpEff 5.0e.^77^ More details about the data analysis are described in Figure S1.

#### T2R4 molecular modeling and ligand docking

To visualize the location of amino acid variants in *TAS2R4* that were significantly associated with S-ECC susceptibility, we used our previous molecular models of T2R4 wild-type and docked with a quorum sensing molecule N-(3-oxododecanoyl) homoserine lactone (3-oxo-C12-AHL).^34^^,^^35^ Previously, both the T2R4 models were validated by extensive site-directed mutagenesis studies and the ligand 3-oxo-C12-AHL which is secreted by Gram-negative bacteria, affects *Candida albicans* morphology, and activates multiple T2Rs.^34^^,^^78^

### Quantification and statistical analysis

A negative binomial Wald test implemented in “DESeq2” R package^79^ was used to detect differentially abundant bacterial and fungal species between the caries-free and S-ECC groups, controlling the false discovery rate (FDR) for multiple comparison. The model was adjusted for sex and batch effect and was done with species level taxa. Batch effect in the microbiome data refers to possible differences between batches due to library preparation and sequencing runs performed in different dates and DNA extraction methods.

To analyze the association between genetic variants in taste genes and S-ECC susceptibility, a case-control association analysis was performed using PLINK. The allele frequency in cases (S-ECC) and controls (caries-free) for each variant was evaluated to determine whether there was a statistical association between the genetic variants and S-ECC status. The differences in allele frequencies were determined using allelic chi-squared (χ^2^) test. Logistic regression with additive genetic model was also used to identify associations between the variants (predictors) and disease status (response), while adjusting for sex and age, in PLINK. Adjusted *p*-values less than 0.05 (corrected for multiple testing by Bonferroni adjustment test) were considered statistically significant.

The association between host genetic variants and the supragingival plaque microbiome was analyzed using linear regression in PLINK v1.9, in an additive genetic model. The non-zero relative abundances of bacteria and fungi were transformed by the natural logarithm and treated as quantitative trait.^80^ The alpha Shannon diversity index was calculated using the R package “phyloseq” (version 1.30.0) and was also used to identify associations between host genetics and microbial diversity. Age, sex, and microbiome sequencing batch were included as covariates. Bacterial and fungal data were analyzed separately.

## Data Availability

• De-identified raw 16S and ITS1 rRNA gene sequences derived from human samples are deposited at NCBI Sequence Read Archive (SRA) Repository and will be publicly available as of the date of publication. Accession number is listed in the key resources table. • All codes are available via open access tools and resources listed in the STAR methods and key resources table. • Any additional information required to reanalyze the data reported in this paper is available from the lead contact upon request.

## Supplemental information

Document S1. Figure S1 and Table S1

Table S2. List of primers used, related to STAR Methods
