# Supplementary material for: Genetic variants in taste genes play a role in oral microbial composition and severe early childhood caries
Source: iScience. 2022 Nov 9;25(12):105489. doi: 10.1016/j.isci.2022.105489 (PMC9668735; doi:10.1016/j.isci.2022.105489)
Supplement: Document S1. Figure S1 and Table S1 [file mmc1.pdf]

iScience, Volume *25*

Supplemental information

Genetic variants in taste genes play a role in oral microbial composition and severe early childhood caries

Vivianne Cruz de Jesus, Betty-Anne Mittermuller, Pingzhao Hu, Robert J. Schroth, and Prashen Chelikani

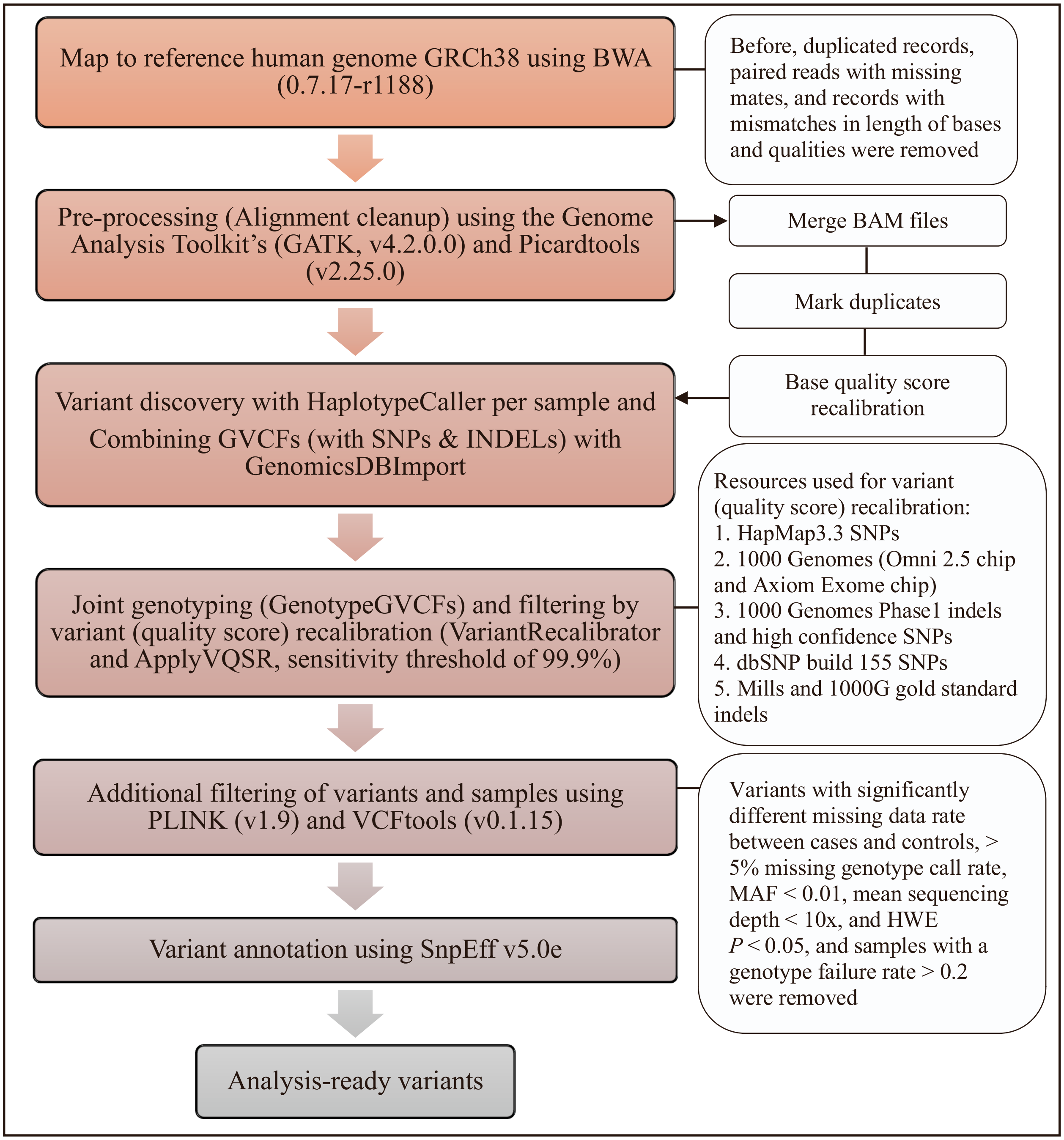

Figure S1. Flow chart of targeted NGS data analysis and quality control. SNPs, single nucleotide polymorphisms, related to STAR Methods. HWE, Hardy–Weinberg equilibrium. MAF, minor allele frequency.

Table S1. Genes sequenced, related to STAR Methods.

Symbol	Gene name	RefSeq ID	Chr	Location (Start-End)	Taste modality
TAS2R1	Taste 2 receptor member 1	NM_019599	5	9627347-9903876	Bitter
TAS2R3	Taste 2 receptor member 3	NM_016943	7	141764097-141765197	Bitter
TAS2R4	Taste 2 receptor member 4	NM_016944	7	141776674-141781691	Bitter
TAS2R5	Taste 2 receptor member 5	NM_018980	7	141790217-141791367	Bitter
TAS2R7	Taste 2 receptor member 7	NM_023919	12	10801532-10802627	Bitter
TAS2R8	Taste 2 receptor member 8	NM_023918	12	10806051-10807286	Bitter
TAS2R9	Taste 2 receptor member 9	NM_023917	12	10809094-10810168	Bitter
TAS2R10	Taste 2 receptor member 10	NM_023921	12	10825219-10826358	Bitter
TAS2R13	Taste 2 receptor member 13	NM_023920	12	10907926-10909562	Bitter
TAS2R14	Taste 2 receptor member 14	NM_023922	12	10937410-10939263	Bitter
TAS2R16	Taste 2 receptor member 16	NM_016945	7	122994704-122995700	Bitter
TAS2R19	Taste 2 receptor member 19	NM_176888	12	11021619-11022620	Bitter
TAS2R20	Taste 2 receptor member 20	NM_176889	12	10995961-10998304	Bitter
TAS2R30	Taste 2 receptor member 30	NM_001097643	12	11132958-11134644	Bitter
TAS2R31	Taste 2 receptor member 31	NM_176885	12	11030387-11031407	Bitter
TAS2R38	Taste 2 receptor member 38	NM_176817	7	141972631-141973773	Bitter
TAS2R39	Taste 2 receptor member 39	NM_176881	7	143183419-143184435	Bitter
TAS2R40	Taste 2 receptor member 40	NM_176882	7	143222037-143223079	Bitter
TAS2R41	Taste 2 receptor member 41	NM_176883	7	143477873-143478796	Bitter
TAS2R42	Taste 2 receptor member 42	NM_181429	12	11185993-11186937	Bitter
TAS2R43	Taste 2 receptor member 43	NM_176884	12	11091287-11092313	Bitter
TAS2R45	Taste 2 receptor member 45	NM_176886	12	291887-292786	Bitter
TAS2R46	Taste 2 receptor member 46	NM_176887	12	11061365-11062294	Bitter
TAS2R50	Taste 2 receptor member 50	NM_176890	12	10985913-10986912	Bitter

TAS2R60	Taste 2 receptor member 60	NM_177437	7	143443453-143444409	Bitter
TAS1R1	Taste 1 receptor member 1	NM_138697	1	6555307-6579755	Umami
TAS1R2	Taste 1 receptor member 2	NM_152232	1	18839599-18859660	Sweet
TAS1R3	Taste 1 receptor member 3	NM_152228	1	1331280-1335320	Umami, Sweet
CA6	Carbonic anhydrase 6	NM_001215.4	1	8,945,868-8,975,092	Carbonation
CA7	Carbonic anhydrase 7	NM_005182.3	16	66,844,414-66,854,147	Carbonation
FFAR1 (GPR40)	Free fatty acid receptor 1	NM_005303.3	19	35,351,552-35,353,862	Fat
FFAR2 (GPR43)	Free fatty acid receptor 2	NM_005306.3	19	35,448,257-35,451,767	Fat
FFAR3 (GPR41)	Free fatty acid receptor 3	NM_005304.5	19	35,358,460-35,360,489	Fat
FFAR4 (GPR120)	Free fatty acid receptor 4	NM_181745.4	10	93,566,665-93,590,072	Fat
OTOP1	Otopetrin 1	NM_177998.3	4	4,188,726-4,226,929	Sour
SCNN1A	Sodium channel epithelial 1 subunit alpha	NM_001159576	12	6,346,843-6,375,224	Salt
SCNN1B	Sodium channel epithelial 1 subunit beta	NM_000336.3	16	23,302,302-23,381,294	Salt
SCNN1G	Sodium channel epithelial 1 subunit gamma	NM_001039.4	16	23,182,745-23,216,883	Salt
SCNN1D	Sodium channel epithelial 1 subunit delta	NM_001130413.4	1	1,280,436-1,292,025	Salt

Note: The starting and ending nucleotide positions are from the human December 2013 (GRCh38/hg38) assembly (http://genome.ucsc.edu). Chr, Chromosome.
